# SOCIOECONOMIC DISADVANTAGE AND EARLY AND RECURRENT ADMISSIONS IN PATIENTS WITH HEART FAILURE

**DOI:** 10.1093/geroni/igae098.3301

**Published:** 2024-12-31

**Authors:** Radha Dhingra, Hanzhang Xu, Bradley Hammill, Scott Lynch, Jessica West, Michael Green, Matthew Dupre

**Affiliations:** Duke University, Durham, North Carolina, United States; Duke University, Durham, North Carolina, United States; Duke University, Durham, North Carolina, United States; Duke University, Durham, North Carolina, United States; Duke University, Durham, North Carolina, United States; Duke University, Durham, North Carolina, United States; Duke University, Durham, North Carolina, United States

## Abstract

The association between socioeconomic status and 30-day readmissions is well-documented in patients with heart failure (HF). However, it is less clear whether socioeconomic disadvantage has an immediate and/or lasting impact on risk of hospital admissions following the diagnosis of HF. We examined whether area-level disadvantage was associated with early and recurrent risks of hospitalizations in patients diagnosed with HF. We included 5,889 patients (ages 65-85 years) with newly diagnosed HF between January 2015 and July 2018 in the Duke University Health System and followed them for up to eight years. We assessed the association of area-level socioeconomic disadvantage (low, moderate, or high) with hospital admissions within 30-days after HF diagnosis using multivariable logistic regression models. We also assessed the risks of recurrent admissions over the entire duration of follow-up using Prentice, Williams, and Peterson models with total time. In our cohort (mean (SD) age: 75 (6) years; 51% female; 67% non-Hispanic White), 71% patients had at least one admission and 40% patients died over a median follow-up of 5.6 years. Area-level disadvantage was not associated with risks of admission within 30-days after HF diagnosis (hazard ratio [95% CI]: HR=1.09 [0.90-1.31]; P=.371). However, patients living in high-disadvantaged areas had significantly greater risks of recurrent admissions over follow-up (HR=1.11 [1.05-1.16]; P<.001). We observed similar patterns for 30-day mortality and mortality over the entire follow-up period. Overall, results from this study suggest that area-level socioeconomic disadvantage should be considered in clinical decision-making and guidelines to improve long-term outcomes in patients managing HF.

